# Search for a small egg by spermatozoa in restricted geometries

**DOI:** 10.1007/s00285-015-0955-3

**Published:** 2015-12-26

**Authors:** J. Yang, I. Kupka, Z. Schuss, D. Holcman

**Affiliations:** Applied Mathematics and Computational Biology, Ecole Normale Supérieure, IBENS, 46 rue d’Ulm, 75005 Paris, France; School of Life and Environmental Sciences, Guilin University of Electronic Technology, Guilin, 541004 Guangxi China; Department of Mathematics, Tel-Aviv University, 69978 Tel-Aviv, Israel; Mathematical Institute, University of Oxford, Andrew Wiles Building, Woodstock Rd, Oxford, OX2 6GG UK

**Keywords:** Narrow escape, Random search process, Spermatozoa, Asymptotic estimates, Numerical simulations, 60H30, 60H35, 92B05

## Abstract

The search by swimmers for a small target in a bounded domain is ubiquitous in cellular biology, where a prominent case is that of the search by spermatozoa for an egg in the uterus. This is one of the severest selection processes in animal reproduction. We present here a mathematical model of the search, its analysis, and numerical simulations. In the proposed model the swimmers’ trajectories are rectilinear and the speed is constant. When a trajectory hits an obstacle or the boundary, it is reflected at a random angle and continues the search with the same speed. Because hitting a small target by a trajectory is a rare event, asymptotic approximations and stochastic simulations are needed to estimate the mean search time in various geometries. We consider searches in a disk, in convex planar domains, and in domains with cusps. The exploration of the parameter space for spermatozoa motion in different uterus geometries leads to scaling laws for the search process.

## Introduction

Searches for a small target by random particle or by random switching dynamics depend critically on the local geometry of the target and the global geometry of the domain, as revealed recently by analytical, numerical methods and by stochastic simulations of molecular motion, viral trafficking, or prey search (Holcman and Schuss 2004; Redner 2001; Benichou et al. 2007; Holcman and Schuss 2013, 2014; Ward et al. 2010; Kurella et al. 2015; Cheviakov et al. 2012; Schuss 2010). Asymptotic formulas for the expected search time were found for diffusive motion (Holcman and Schuss 2013), but much less is known for other search processes. The problem under consideration here is that of estimating the mean search time of spermatozoa for an egg lodged in the uterus or in the fallopian tubes. This search is one of the most severe selection processes of one among hundreds of millions spermatozoa. How the winner is selected remains unclear and may depend on many parameters of the spermatozoa and the uterus. It is also unclear how to quantify geometrical parameters of the uterus. For example, how does a drop in the initial number of sperms affect fertility?

Between 1989 and 2005 the concentration of sperm cells in human semen has dropped significantly and continuously at an average rate of 1.9 %/year, leading to a reduction of 32.2 % in sperm count over 16 years (Rolland et al. 2013). Do these changes in concentration really matter? It is well documented that reducing the concentration of spermatozoa by four leads to infertility, but how such a result can be explained? because it still remains millions Reynaud et al. (2015). We still do not understand the role of the huge redundancy in sperm population. Motivated by these questions, we develop here a mathematical model of spermatozoa motion based on a simplified dynamics and on some elements of the uterus geometry. Our goal is to estimate the expected arrival time of a spermatozoon (a sperm cell) to a given neighborhood of a small egg. In that neighborhood, the motion of a spermatozoon is modified, because it responds to chemotaxis gradient generated by the egg (Armon et al. 2014; Armon and Eisenbach 2011; Teves et al. 2009). This terminal phase of the search is not studied here.

Classical models of spermatozoa motion include the beating of flagella in viscoelastic fluids (Fu et al. 2008) and attraction to a flat wall due to hydrodynamic interactions of the swimmer with the surface (Elgeti et al. 2010; Berke et al. 2008; Smith et al. 2011; Gaffney et al. 2011; Kantsler et al. 2014, 2013). For high spermatozoa concentration, collective modes of locomotion, different from those displayed by isolated cells, have been described by long-time kinematics of their relative locomotion (Michelin and Lauga 2010). The study of asymmetric flagellar bending was based on cytoplasmic calcium dynamics in the flagellum (Olson et al. 2011). The trajectories of spermatozoa can also be influenced by fluid motion (Marcos et al. 2012). However none of the present studies have addressed the generic question of the search of a small egg in the context of the uterus. Indeed, we shall explore here the role of the uterus geometry, quantified by various parameters such as the height, width, the radius of the cervix $\delta$, local curvature near the fallopian tubes or the aspect $\rho =L/W$ (as shown in Fig. 4) that measures the non-convexity, all should be key parameters in directing the spermatozoa toward the egg, yet this possibility has not been explored so far. However, contrary to the mentioned references, in the present work, we will use a very crude model of the spermatozoa motion, approximated as a ballistic directed motion, to derive asymptotic formula for the search process in convex geometries and use numerical simulations for non-convex ones. The novelty and difficulty here are in geometry of the uterus-like domain, where the motion occurs and the search for the small egg target.

**Fig. 1:**
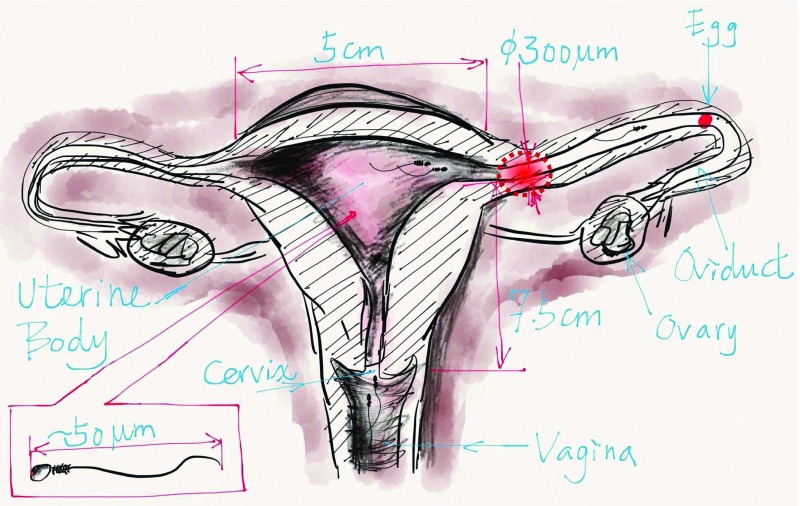
Schematic representation of the human uterus, showing the fallopian tubes, ovaries, cervix, vagina, and the ovum. We emphasize various location of the target (*round dashed circle*)

We recall that the adult female uterus (Gray et al. 1974) is pear-shaped and is about 7.5 cm long, has a maximum diameter of 5 cm and a height of 3.4 cm, with a mean volume of tens of $\mathrm{cm}^3$. It is a hollow thick-walled non-convex muscular organ. On its upper part, the uterine tubes open, one on either side, while below, its cavity connects to that of the vagina. After an egg is released from the ovaries and moves inside the uterine cavity through the uterine tubes, it waits for fertilization (see Fig. 1). In summary, fertilization occurs most likely between the junction at the end of the uterus and somewhere in the fallopian tube, but not inside the uterus. We thus define the position of the target for this search process as the entrance of the fallopian tube, modeled here as a small gap between straight line and quarter-ellipses (see Fig. 1).

In estimating the expected search time, we model the spermatozoa motion as rectilinear far away from the egg, with random reflection when a trajectory hits the boundary of the uterus. Indeed, we adopt this model here following recent in vivo movies that we observed (courtesy of Reynaud K, private communication), where spermatozoa motions were mostly ballistic for distances of millimeters, as long as they do not encounter any obstacle. After hitting a small obstacle, the new direction was much correlated to the previous one, which is a significant deviation from a classical random walk. However, after hitting a wall and for trajectories that immediately returned to the bulk, the reflected angle was not correlated with the initial one. Other reflection behaviors include motion along the boundary, as observed in microfluidic chambers (Denissenko et al. 2012). It is however unclear at this time what exactly is the spermatozoa motion in vivo at long distances of centimeter sizes. We further construct numerical simulations of the search to explore a part of the phase-space, especially for non-convex shape geometry, where we have not yet found a fruitful analytical approach. Mathematically, finding a small target by this search process with deterministic reflection is analogous to the escape from a billiard table through one and two small holes (Bunimovich and Dettmann 2005).

This manuscript is organized as follows. We start with describing a crude rectilinear model for the swimmer motion and use it to estimate the time to find a small target (one of the two small fallopian entrances, which are two well-separated small targets, see Fig. 1). The swimmer motion is modeled as that of a point moving at constant speed in a fixed direction. It is reflected at the boundary in a random direction and absorbed when it hits the small target (of size $\varepsilon$). We estimate analytically the arrival time to the small target for different situations: starting with the unit disk, followed by the three-dimensional ball and then for more general two-dimensional convex domains (that are small deviation of a disk). We explore non-convex geometries by numerical simulations in dimension two. For non-convex domains, there are regions, including the one where the target is located, which are not accessible to direct rays, leading to a difficulty in computing the probability to find the target in finite time. The present approach provides a framework for the study of various parameters involved in the search process and reveals the role of the geometry in defining the search time.

## A crude model of spermatozoa motion

### Model simplifications

Spermatozoa motion results from the flagella beating attached to the round head. The ensemble head plus flagella allows the spermatozoa to swim in a complex medium, inside or on the surface of a domain. At a micrometer scale, the details of the spermatozoa geometry are relevant to the prediction of its motion, to account for the variety of displacements and of its orientation. For example, spermatozoa are reflected along a corner or at a surface with different properties, with random angles that are different from the mechanical and optical rules of the Snell-Descartes law of reflection (Kantsler et al. 2013; Ishimoto and Gaffney 2014).

In the present study, we focus on long-scale motion on the order of centimeters. We neglect the effect of any chemotaxis gradient that could direct the cell toward a specific target. Thus the motion of a spermatozoon is simplified to that of a point, which away from any boundary, is ballistic or rectilinear with a constant velocity (Prez-Cerezales et al. 2015). We postulate that when a spermatozoon hits a surface it is reflected at a random angle, uniformly distributed in the tangent half-space. Thus the simplified model of spermatozoon motion in a bounded domain $\Omega$ is assumed rectilinear with constant velocity

1 $$\begin{aligned} \dot{\varvec{X}}=v_0 \varvec{u}, \end{aligned}$$

where $\varvec{u}$ is a fixed vector chosen on the unit sphere from a uniform distribution. Upon hitting an obstacle at a boundary point $\varvec{X}_0$, the velocity changes to

2 $$\begin{aligned} \dot{\varvec{X}}=v_0 \varvec{v}, \end{aligned}$$

where $\varvec{v}$ is chosen on the unit sphere in the supporting half space at $\varvec{X}_0$ from a uniform distribution, independently of $\varvec{u}$. This model captures the crude sperm dynamics, neglecting the additional motion induced by the flagella and will be implemented in the simulations performed here.

## The expected search time in convex domains

### Expected search time in a disk

When $\Omega$ is a planar circular disk of radius *R*, the velocity is $v_0$, and the target is an arc of length $\varepsilon$ on the boundary, the probability $p(\varepsilon )$ to hit the target in one step, starting the search at any point on the circle, is

3 $$\begin{aligned} p(\varepsilon )=\frac{\varepsilon }{2\pi R}. \end{aligned}$$

**Fig. 2:**
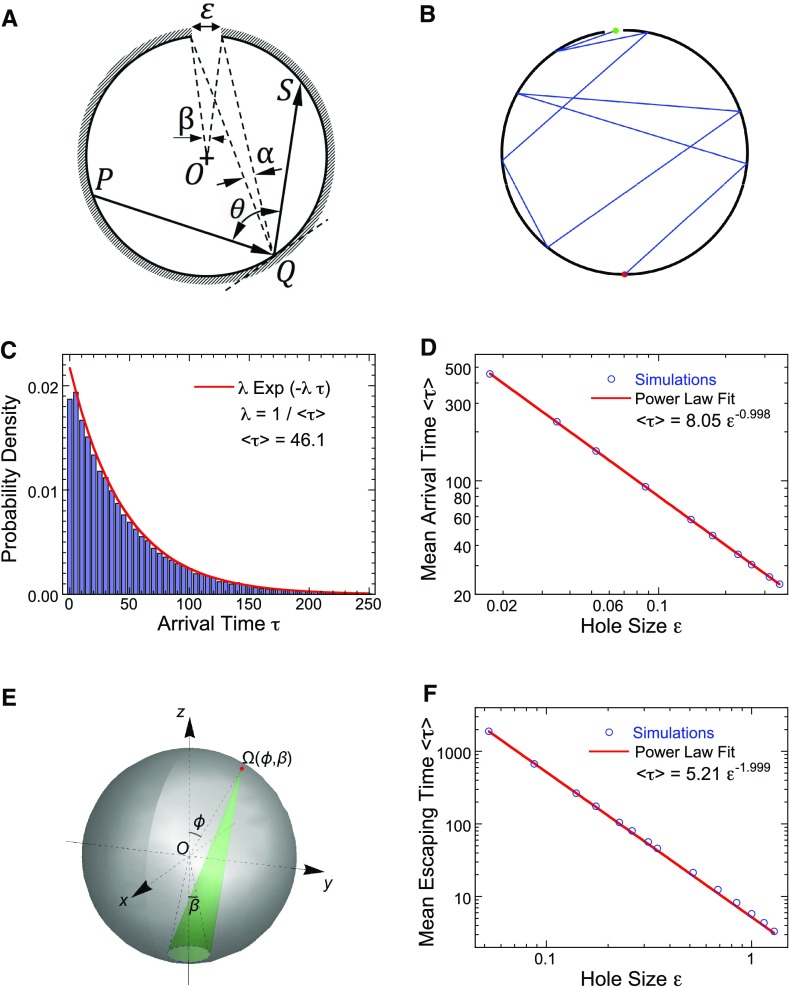
Search for a small target in a disk and a ball. **a** Schematic representation of the dynamics in a disk: the motion is reflected on the boundary at a random angle. The target size $\varepsilon$ is on the boundary. **b** A path with many random reflection escapes ultimately at the target (*green dot*). **c** Probability density function of the arrival time obtained from stochastic simulations. **d** The expected search time is the MFPT of the trajectory to the target, obtained from stochastic simulations (*blue circles*) and compared to the analytical (9) (*red*). **e** Search in a ball. **f** Comparison of the analytical curve (*red*) with the stochastic simulations (*blue circles*). Non-dimensional parameters: $R=1$, $v_0=1$ (color figure online)

The probability to hit the target in exactly *k* steps is a geometric distribution $q_k=(1-p(\varepsilon ))^{k-1}p(\varepsilon )$. Thus the expected number of steps to the target is

4 $$\begin{aligned} {\mathbb {E}}[N_{hit}] = \sum \limits _{k=0}^{\infty } kq_k=\frac{1}{p(\varepsilon )}=\frac{2\pi R}{\varepsilon }. \end{aligned}$$

To determine the expected search time, we assume that the target is centered at $x=0,\,y=R$ and that *k* reflections occur at the uniformly distributed angles $(\theta _1,\ldots \theta _k)$ (Fig. 2), where the angle of reflection is $\theta =\widehat{PQS}$. Each ray is represented by its projection $Re^{i\theta _k}$ on the circle of radius R. The distance between the reflection points is

5 $$\begin{aligned} d_k=2R\left| \sin \frac{\theta _k-\theta _{k-1}}{2}\right| . \end{aligned}$$

The expectation of the right hand side is given by

6 $$\begin{aligned} {\mathbb {E}}_{\theta _k,\theta _{k-1}}\left[ \left| \sin \frac{\theta _k-\theta _{k-1}}{2}\right| \right]= & {} \int _{0}^{2\pi } \int _{0}^{2\pi }\left| \sin \frac{\theta _k-\theta _{k-1}}{2}\right| \frac{d\theta _k}{2\pi }\frac{d\theta _{k-1}}{2\pi }\end{aligned}$$

7 $$\begin{aligned}= & {} 2\int _{0}^{\pi /2}\left| \sin \frac{\theta }{2} \right| \frac{d\theta }{\pi }=\frac{2}{\pi }. \end{aligned}$$

The time spent on a single ray is $\tau _k=d_k/v_0$, therefore the mean time to exit in *N* steps is

8 $$\begin{aligned} \mathbb {E}[\tau \,|\,N]=\frac{1}{v_0}\mathbb {E}\sum _{k=1}^{N}2R \left| \sin \frac{\theta _k-\theta _{k-1}}{2}\right| =\frac{4RN}{\pi v_0}. \end{aligned}$$

Thus the mean first passage time to the target is

9 $$\begin{aligned} \mathbb {E}[\tau ]=\sum _{N=1}^\infty \mathbb {E}[\tau \,|\,N](1-p(\varepsilon ))^{N-1}p(\varepsilon )=\frac{4R}{\pi v_0 p(\varepsilon )}=\frac{8R^2}{ v_0 \varepsilon }=\frac{8S}{\pi v_0 \varepsilon }, \end{aligned}$$

where $S=\pi R^2$. Formula (9) shows that the present search process is asymptotically of the order $\frac{1}{\varepsilon }$, as $\varepsilon$ goes to zero, much longer than the one for a free Brownian particle with diffusion coefficient *D*, searching for a target of size $\varepsilon$, in a two-dimensional domain of surface *S*, for which the Narrow escape theory (Cheviakov et al. 2012; Schuss et al. 2007) gives

10 $$\begin{aligned} \mathbb {E}[\tau ]\approx \frac{S}{\pi D} \log \frac{1}{\varepsilon } +O(1). \end{aligned}$$

The results of numerical simulations of a search for a small arc (Fig. 2a) in a circular disk are shown in Fig. 2b, where the search starts at a point uniformly distributed on the boundary (red point). The tail of the exit time density decays exponentially (Fig. 2c). The increase at short times corresponds to a fraction of trajectories that shoot-up directly to the target. Finally, the numerical approximation for the expected search time is well matched by the asymptotic formula (9), where

$$\begin{aligned} \mathbb {E}[ \tau ] \sim \frac{1}{\varepsilon } \end{aligned}$$

(Fig. 2d, where for the simulations, we took non dimensional parameters $R=1$, $v_0=1$).

The distribution of the number of search steps prior to hitting the target decays exponentially, because

11 $$\begin{aligned} p_k=\Pr \{ N=k\}=(1-p(\varepsilon ))^{k-1}p(\varepsilon )=p(\varepsilon ) \exp \{ (k-1)\log (1-p(\varepsilon )) \}. \end{aligned}$$

Thus, the probability density of the search time $\tau _e$ is

12 $$\begin{aligned} \ \Pr \{\tau _e= t\}=\sum _k \Pr \{\tau _e= t\,|\,k\} p_k \quad \text{ for } t \gg 1. \end{aligned}$$

The exit time event $\{\tau _e= t\}$ can always be decomposed as $\tau _e=k\tau _{m}+t^*$, where the residual time $t^*$ is distributed as

13 $$\begin{aligned} \Pr \{t^*=s\} = \left\{ \begin{array}{ll} \alpha s, &{} s\le \tau _{m}/2 \\ \alpha (\tau _{m}-s), &{} \tau _{m}/2\le s< \tau _{m}. \end{array}\right. \end{aligned}$$

where $\tau _{m}=2R/v_0$ (the maximum search time) and the normalization constant is $\alpha = \frac{1}{\int _{0}^{\tau _{m}}\Pr \{t^*=s\}ds}$. Indeed the velocity is constant and the initial point is uniformly distributed on the circle. Thus, as the number of steps before exit decays exponentially, we have that

14 $$\begin{aligned}&\Pr \{\tau _e= t\}=\int _{0}^{\tau _{m}} \Pr \{\tau _e= k\tau _{m}+t^*=t, t^*=s\} \Pr \{t^*=s\}ds, \end{aligned}$$

15 $$\begin{aligned}&\Pr \{\tau _e = k\tau _{m}+t^*=t, t^*=s\} \sim \Pr \{\tau _e= k\tau _{m} = t-s\} \sim \Pr \{\tau _e = k\tau _{m}=t\} \hbox { for } t\gg 1.\nonumber \\ \end{aligned}$$

Thus,

16 $$\begin{aligned} \Pr \{\tau _e= t\} \sim \exp (-\lambda _\varepsilon t), \end{aligned}$$

where

17 $$\begin{aligned} \lambda _\varepsilon =\log \frac{1}{(1-p(\varepsilon ))}\approx p(\varepsilon ). \end{aligned}$$

### Expected search time in the three-dimensional ball

To compute the expected search time of a swimmer in a ball, we assume that the target is a small disk centered at the north pole of the sphere and that the dynamics are (1) and (2). The apex of the target is an angle $\beta =2\varepsilon$ on the sphere, the solid angle at any point on the sphere at position $\phi$ is in general a function of both variables $\phi$ and $\varepsilon$, denoted $\Omega (\phi ,\varepsilon )$ (see Fig. 2), $\theta , \phi$ are spherical coordinates on the sphere.

At each step of hitting the boundary, the probability for the trajectory to find the target is $p(\theta )=\Omega (\phi ,\varepsilon )/2\pi$. The solid angle $\Omega (\phi ,\varepsilon )$ depends on the position and the size of the hole (Paxton 1959) and the exact expression for formula (62) is given in Appendix A.2. However, in contrast to the case of a disk, the expected number of steps to hit the target, or the search time, depends on the history, due to the dependence of the probability on the variable $\phi$. Indeed, the probability to hit the target in *k* steps, with the history angles $\phi _{1},..,\phi _{k}$, is

18 $$\begin{aligned} p\left( \phi _{k},\varepsilon \right) \prod \limits _{i=1}^{k-1} \left[ 1-p\left( \phi _{i},\varepsilon \right) \right] . \end{aligned}$$

Thus the expected number of hits prior to escape is

19 $$\begin{aligned} \mathbb {E}[N_{hit}] = \sum \limits _{k=1}^{\infty } k\, \mathbb {E}\left[ p\left( \phi _{k},\varepsilon \right) \prod \limits _{i=1}^{k-1} \left[ 1-p\left( \phi _{i},\varepsilon \right) \right] \,{\Big |}\,\phi _{1},..,\phi _{k}\right] , \end{aligned}$$

where the expectation is conditioned on all trajectories that are reflected with angles $(\phi _{1},..,\phi _{k-1})$, which are uniformly distributed in $[0,\pi -\varepsilon ]$, while the last angle falls into $[\pi -\varepsilon ,\pi ]$. Thus, because all angles variables are i.i.d. random variables

20 $$\begin{aligned} \mathbb {E}[N_{hit}]=\sum \limits _{k=1}^{\infty } k\, \mathbb {E}p\left( \phi ,\varepsilon \right) \left[ 1-\mathbb {E}p\left( \phi ,\varepsilon \right) \right] ^{k-1} \sim \frac{1}{\mathbb {E}p\left( \phi ,\varepsilon \right) }=\frac{2 \pi }{ \langle \Omega \left( \phi ,\varepsilon \right) \rangle },\qquad \end{aligned}$$

where the mean solid angle is defined by

21 $$\begin{aligned} \langle \Omega \left( \phi , \varepsilon \right) \rangle = \dfrac{\int _{0}^{2 \pi }\int _{0}^{\pi -\varepsilon }\Omega \left( \phi ,\varepsilon \right) \sin \phi \, d \phi \, d \theta }{\int _{0}^{2\pi }\int _{0}^{\pi -\varepsilon }\sin \phi \, d\, \phi \, d \theta }=\dfrac{\int _{0}^{\pi -\varepsilon }\Omega \left( \phi ,\varepsilon \right) \sin \phi \,d \phi }{1+\cos \varepsilon }. \end{aligned}$$

The solid angle at which the target is seen from the sphere center is $\Omega _{0}=2\pi (1-\cos \varepsilon )$. Note that $\lim _{\varepsilon \rightarrow 0}\Omega _{0} / \langle \Omega ( \phi , \varepsilon )\rangle =2$ (see Appendix A.2).

To calculate the expected search time, we first compute the mean Euclidean distance along a ray between two consecutive reflecting points $P_{k-1}=(\phi _{k-1},\theta _{k-1})$ and $P_k=(\phi _k,\theta _k)$ on the sphere, where $(\phi , \theta )$ are the spherical angles uniformly distributed in $[0,2\pi ]\times [0,\pi ]$. First, on a ball of radius *R*,

$$\begin{aligned} d(P_{k},P_{k-1})^2&=R^2(\left( \sin \theta _{k}\cos \phi _{k}-\sin \theta _{k-1}\cos \phi _{k-1}\right) ^{2}\\&\quad +\left( \sin \theta _{k}\sin \phi _{k}-\sin \theta _{k-1}\sin \phi _{k-1}\right) ^{2}\\&\quad +\left( \cos \theta _{k}-\cos \theta _{k-1}\right) ^{2} )\\&=2R^2(1-\sin \theta _{k-1}\sin \theta _{k}\cos (\phi _{k}-\phi _{k-1})). \end{aligned}$$

Thus,

22 $$\begin{aligned} d_{S}=\langle d(P_{k},P_{k+1})\rangle&= \frac{\sqrt{2}R}{4 \pi ^{4}}\int _{0}^{2\pi }d\phi _{k-1}\int _{0}^{2\pi }d\phi _{k}\int _{0}^{\pi }d\theta _{k-1} \int _{0}^{\pi }d\theta _{k}\nonumber \\&\quad \times \sqrt{(1-\sin \theta _{k-1}\sin \theta _{k}\cos (\phi _{k}-\phi _{k-1}))}. \end{aligned}$$

The integral (22) is evaluated numerically (Mathematica) to give an approximation for the mean length on a sphere

23 $$\begin{aligned} d_{S}=\langle d(P_{k},P_{k+1})\rangle \approx 1.32\, R. \end{aligned}$$

It follows that the expected hitting time after exactly *N* hits is

$$\begin{aligned} \mathbb {E}[\tau \,|\,N]=\frac{1}{v_0}\mathbb {E}\left[ \sum _{k=1}^{N}d(P_{k},P_{k+1})\right] =\frac{1.32 RN}{ v_0}, \end{aligned}$$

where $v_{0}$ is the moving speed of the swimmer, which is set to a constant value here. Using (19), we finally get the general formula for the expected search time

24 $$\begin{aligned} \mathbb {E}[\tau ]=\sum \limits _{k=1}^{\infty } \mathbb {E}[\tau \,|\,k ]\mathbb {E}p\left( \phi ,\varepsilon \right) \left[ 1-\mathbb {E}p\left( \phi ,\varepsilon \right) \right] ^{k-1}=\frac{d_{S}}{v_{0}}\frac{2 \pi }{\langle \Omega \left( \phi , \varepsilon \right) \rangle }. \end{aligned}$$

Hence using expression (21), it follows the estimate

25 $$\begin{aligned} \mathbb {E}[\tau ]\approx \frac{2.64R}{v_{0}\left( 1-\cos \varepsilon \right) }. \end{aligned}$$

Figure 2 compares the analytical approximation (25) with results of simulations. The comparison confirms that the leading order of magnitude in the expansion of the expected search time in powers of $\varepsilon$ is $\varepsilon ^{-2}$. A numerical fit gives $\mathbb {E}[\tau ]=2.61 (1-\cos \varepsilon )^{-1.002}$ ($R=1$), hence the expected search time for the target in the three-dimensional ball is proportional to the surface area and inversely proportional to the square radius of the small target (relation 25). An asymptotic formula for an elliptic target can be derived from formula 62 (Appendix A.2) involving elliptic integrals.

### Expected search time in a two-dimensional convex domain

Next, we consider the search for a small target in a planar starlike domain $\Omega$. We calculate the probability of a swimmer’s trajectory to hit a small target on the boundary $\partial \Omega$ of the domain, which is parameterized in polar coordinates by $r(\theta )$ so that in the complex plane, $Q(\theta )=r(\theta ) e^{i \theta }$.

#### The probability to hit a small window

**Fig. 3:**
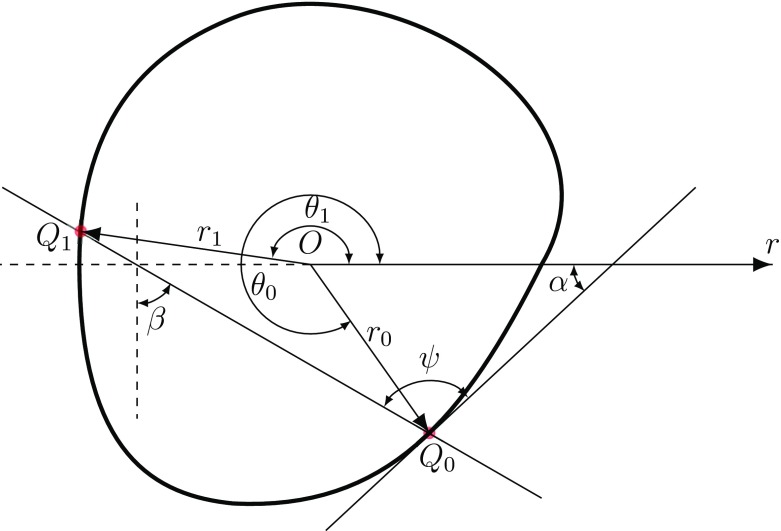
Particle moving from point $Q_{0}$ to $Q_{1}$

When a trajectory starts at $Q_{0}\in \partial \Omega$ it ends at another point $Q_{1}\in \partial \Omega$ (see Fig. 3), the probability that the angular deviation of the particle moving path $Q_{0}Q_{1}$ (assuming the path is a straight line) will lie between angle $\psi$ and $\psi +d\psi$ is $P(dQ_{1}|Q_{0})=d\psi /\pi$, in which $\psi$ is the angle between the moving path and the tangent line at point $Q_{0}$.

Based on triangle geometry in the plane, we obtain $\pi /2+\beta =\psi +\alpha$, in which $\beta$ is the angle between the line $Q_{0}Q_{1}$ and the vertical direction (see Fig. 3) and $\alpha$ is the angle of the tangent line at $Q_{0}$, then it turns out that $d\psi =d\beta$ after applying differentiation to the equation ($\alpha$ is constant). The line $Q_{0}Q_{1}$, can be written in polar coordinate.

$$\begin{aligned} r\cos (\theta -\beta )={{\mathrm {constant}}}, \end{aligned}$$

in which $(r,\theta )$ are polar coordinates in the plane. Therefore the coordinates of the points $Q_{0}=(r_{0},\theta _{0})$ and $Q_{1}=(r_{1},\theta _{1})$ satisfy the equation

26 $$\begin{aligned} r_{0}\cos \left( \theta _{0}-\beta \right) =r_{1}\cos \left( \theta _{1}-\beta \right) , \end{aligned}$$

where $r_{0}=r (\theta _{0})$ and $r_1=r (\theta _{1})$. Expanding (26) and rearranging, we obtain

27 $$\begin{aligned} \tan \beta = \frac{r_{1}\cos \theta _{1}-r_{0}\cos \theta _{0}}{r_{0}\sin \theta _{0}-r_{1}\sin \theta _{1}}=\frac{N(\theta _{1})}{D(\theta _{1})}. \end{aligned}$$

Differentiating (27), we get

28 $$\begin{aligned} \left( 1+\tan ^{2}\beta \right) \frac{d\beta }{d\theta _{1}}=\frac{1}{D(\theta _{1})^{2}}\left( D(\theta _{1}) \frac{dN(\theta _{1})}{d\theta _{1}}-N(\theta _{1}) \frac{dD(\theta _{1})}{d\theta _{1}}\right) , \end{aligned}$$

hence

$$\begin{aligned} d\beta = \frac{1}{D^{2}+N^{2}}\left( D\frac{dN}{d\theta _{1}}-N \frac{dD}{d\theta _{1}}\right) d\theta _{1}= \frac{r_1^2-r_0\displaystyle \frac{d}{d\theta _1}\left[ r_1\sin (\theta _1-\theta _0)\right] }{r_1^2+r_0^2-2 r_1 r_0\cos \left( \theta _1-\theta _0\right) }d\theta _1. \end{aligned}$$

Therefore, the probability density of hitting the target in one step, $P(dQ_{1}|Q_{0})$, can be written as

29 $$\begin{aligned} P\left( dQ_{1}|Q_{0}\right) =\frac{d\psi }{\pi }=\frac{d\beta }{\pi }= \frac{r_{1}^{2}-r_0\dfrac{d}{d\theta _{1}}\left[ r_{1}\sin \left( \theta _{1}-\theta _{0}\right) \right] }{\pi \left( r_{1}^{2}+r_{0}^{2}-2 r_{1} r_{0}\cos \left( \theta _{1}-\theta _{0}\right) \right) }d\theta _{1}. \end{aligned}$$

In contrast to the case of a circle, here the density depends on the initial position.

#### The probability of hitting the target after *n* steps

The probability for a swimmer starting at $Q_{0}\in \partial \Omega$ to hit a small arc $\partial \Omega _a$ of length $|\partial \Omega _a|=\varepsilon$, centered at $Q_{1}\in \partial \Omega _a$, is

30 $$\begin{aligned} P_{1}(Q_{0})=\int \limits _{\partial \Omega _a}P\left( dQ_{1}|Q_{0}\right) . \end{aligned}$$

The probability to hit the target after bouncing *n* times in the boundary $\partial \Omega _r=\partial \Omega -\partial \Omega _a$ is

31 $$\begin{aligned} P_{n}(Q_{0})=\int \limits _{\partial \Omega _a}\underbrace{\int \limits _{\partial \Omega _r} \cdots \int \limits _{\partial \Omega _r}}_{n-1}P(dQ_{n}|Q_{n-1})P(dQ_{n-1}|Q_{n-2})\cdots P(dQ_{1}|Q_{0}). \end{aligned}$$

There is no general simplifying form for this multiple integral. We consider, therefore, a perturbation of a circle.

#### Perturbation of a circle

A perturbation of the unit circle can be written as

32 $$\begin{aligned} r(\theta )=1+\eta \rho (\theta ), \end{aligned}$$

which for $\eta \ll 1$ is a small amplitude deviation, as long as $\rho$ is a function that keeps the domain convex. Substituting (32) in (29) and expanding to leading order in powers of $\eta$ we obtain

33 $$\begin{aligned} P(dQ_{1}|Q_{0})=\frac{1}{2\pi } \left[ 1+ \frac{\eta }{1-\cos \left( \theta _{1}-\theta _{0}\right) } \left( -\rho _{0}+\rho _{1} -\dfrac{d\rho _{1}}{d\theta _{1}}\sin \left( \theta _{1}-\theta _{0}\right) \right) \right] d\theta _{1} \qquad \quad \end{aligned}$$

which allows to approximate the exit probability after n$^{th}$ iterations (relation (31)) as

34 $$\begin{aligned} P_n(Q_0)= & {} \left( \frac{1}{2\pi }\right) ^{n+1} \int \limits _{\partial \Omega _a}\underbrace{\int \limits _{\partial \Omega _r}\cdots \int \limits _{\partial \Omega _r}{}}_{n-1} \Bigg [ 1+\eta \sum _{k=1}^n\frac{1}{1-\cos \left( \theta _{k}-\theta _{k-1}\right) } \bigg (-\rho _{k-1}+\rho _{k} \nonumber \\&-\dfrac{d\rho _{k}}{d\theta _{k}}\sin \left( \theta _{k}-\theta _{k-1}\right) \bigg )\Bigg ]\,d\theta _1\cdots \,d\theta _n. \end{aligned}$$

For $$\eta \ll 1$$ and $$n\ge 2$$

35 $$\begin{aligned} P_{n}(Q_0)= \left( \frac{1}{2\pi }\right) ^{n} \varepsilon (2\pi -\varepsilon )^{n-1}+ \eta \tilde{P}_{n}(\varepsilon ) +O(\eta ^2), \end{aligned}$$

and

36 $$\begin{aligned} P_1(Q_0)= \left( \frac{\varepsilon }{2\pi }\right) +\eta \tilde{P}_{1}(\varepsilon |\theta _0) +O(\eta ^2), \end{aligned}$$

where

37 $$\begin{aligned} \tilde{P}_{n}(\varepsilon |\theta _0)&= \left( \frac{1}{2\pi }\right) ^{n}\int \limits _{\partial \Omega _a}\underbrace{\int \limits _{\partial \Omega _r}\cdots \int \limits _{\partial \Omega _r}}_{n-1} \sum _{k=1}^n\frac{d\theta _1\cdots d\theta _{n+1}}{1-\cos \left( \theta _{k}-\theta _{k-1}\right) }\nonumber \\&\quad \times \left( -\rho _{k-1}+\rho _{k} -\dfrac{d\rho _{k}}{d\theta _{k}}\sin \left( \theta _{k}-\theta _{k-1}\right) \right) . \end{aligned}$$

This can be further simplified to

$$\begin{aligned} \tilde{P}_{n}(\varepsilon |\theta _0)= Q_{n}(\varepsilon )+ R_{n}(\varepsilon )+ T_{n}(\varepsilon ) , \end{aligned}$$

with

$$\begin{aligned} Q_{n}(\varepsilon )&= (n-1)\left( \frac{1}{2\pi }\right) ^{n-1}|\partial \Omega _a||\partial \Omega _e|^{n-2} K_1(f_{\rho })\quad \hbox {for } n \ge 2\\ R_{n}(\varepsilon )&=\left( \frac{|\partial \Omega _r|}{2\pi }\right) ^{n-1} K_2(f,\partial \Omega _a)\quad \hbox {for } n \ge 1\\ T_{n}(\varepsilon |\theta _0)&=\left( \frac{|\partial \Omega _r|}{2\pi }\right) ^{n-1}\frac{|\partial \Omega _a|}{4\pi ^2}\int \limits _{\partial \Omega _r}P(dQ_{1}|\theta _{0}). \end{aligned}$$

where

38 $$\begin{aligned} K_1(f_{\rho })&= \frac{1}{4\pi ^2}\int \limits _{\partial \Omega _r}\int \limits _{\partial \Omega _r} f_{\rho }(\theta _{1},\theta _{2})\,d\theta _1\,d\theta _2 \end{aligned}$$

39 $$\begin{aligned} K_2(f_{\rho },\partial \Omega _{a})&=\frac{1}{4\pi ^2}\int \limits _{\partial \Omega _r}\int \limits _{\partial \Omega _a} f_{\rho }(\theta _{1},\theta _{2})\,d\theta _1\,d\theta _2\end{aligned}$$

40 $$\begin{aligned} f_{\rho }(\theta _{1},\theta _{2})&=\frac{1}{1-\cos \left( \theta _{2}-\theta _{1}\right) } \left( -\rho _{1}+\rho _{2} -\dfrac{d\rho _{2}}{d\theta _{2}}\sin \left( \theta _{2}-\theta _{1}\right) \right) . \end{aligned}$$

The operators $K_1(f_{\rho })$ and $K_2(f_{\rho },\partial \Omega _a)$ are well defined, because

41 $$\begin{aligned} \lim _{\theta _{2}\rightarrow \theta _{1}} f_{\rho }(\theta _{1},\theta _{2})=-\rho _{1}''. \end{aligned}$$

#### Number of hits before hitting the target

The expected number of steps $\mathbb {E}[N_{hit}]$ before hitting the target is expressed in terms of the probability $P_{n}(Q_{0})$ to hit the target in *n*-steps, given the initial point $Q_0$,

42 $$\begin{aligned} \mathbb {E}[N_{hit}] = \sum _{1}^{\infty } k P_{k-1}(Q_{0}). \end{aligned}$$

The expansion (34) for small $\varepsilon$ and $\eta$ gives to leading order

43 $$\begin{aligned} \mathbb {E}[N_{hit}] = \sum _{1}^{\infty } k P_{k-1}(Q_{0})=\frac{2\pi }{\varepsilon }+ \eta \sum _{1}^{\infty } k \tilde{P}_{k-1}(\varepsilon |\theta _0). \end{aligned}$$

We decompose

44 $$\begin{aligned} \sum _{1}^{\infty } k \tilde{P}_{k-1}(\varepsilon |\theta _0)=Q(\varepsilon )+R(\varepsilon )+T(\varepsilon |\theta _0), \end{aligned}$$

with

$$\begin{aligned} Q(\varepsilon )&=\sum _{2}^{\infty } nQ_{n-1}(\eta )= 2\frac{(2\pi )^2}{\varepsilon ^2} K_1(f_{\rho }) \\ R(\varepsilon )&=\sum _{1}^{\infty } nR_{n-1}(\eta )=\frac{4\pi ^2}{\varepsilon ^2}K_2(f,J^c)\\ T(\varepsilon \,|\,\theta _0)&=\sum _{1}^{\infty } n\tilde{T}_{n}(\varepsilon )=\frac{1}{\varepsilon } \int _{J}P(dQ_{1}\,|\,\theta _{0}) \end{aligned}$$

to obtain for $\eta \ll \varepsilon$,

45 $$\begin{aligned} \mathbb {E}[ N_{hit}] = \frac{2\pi }{\varepsilon }+ 2\eta \frac{4\pi ^2}{\varepsilon ^2} K_1(f_{\rho })+ O\left( \frac{\eta ^2}{\varepsilon ^2}\right) . \end{aligned}$$

It follows that the expected number of steps prior to hitting the target depends on the integral $K_1(f_{\rho })$, which accounts for certain global properties of the boundary.

To compute the mean time before escape, we estimate the distance between two points $P(\theta _1)$ and $P(\theta _2)$ using the perturbation condition (32)

$$\begin{aligned} d_{12}(\theta _1,\theta _2)&= |P(\theta _1)P(\theta _2)|= |r(\theta _1)e^{i\theta _1}-r(\theta _2)e^{i\theta _2}|\\&=|e^{i\theta _1}-e^{i\theta _2}| +\frac{\eta }{2|e^{i\theta _1}-e^{i\theta _2}|}(\rho _1+\rho _2)(1-\cos (\theta _1+\theta _2))+O(\eta ^2)\\&= 2\left| \sin \frac{\theta _1-\theta _2}{2}\right| +\frac{\eta (\rho _1+\rho _2)}{2}\left| \sin \frac{\theta _1-\theta _2}{2}\right| +O(\eta ^2). \end{aligned}$$

Thus,

$$\begin{aligned} \mathbb {E}[d_{12}]&= \frac{1}{4\pi ^2}\int \limits _{0}^{2\pi } \int \limits _{0}^{2\pi } 2\left| \sin \frac{\theta _1-\theta _2}{2}\right| +\frac{\eta (\rho _1+\rho _2)}{2}\left| \sin \frac{\theta _1-\theta _2}{2}\right| \,d\theta _1d\theta _2 +O(\eta ^2) \\&= \frac{4}{\pi } + \frac{\eta }{4\pi ^2} \int \limits _{0}^{2\pi }\int \limits _{0}^{2\pi }\frac{\rho _1+\rho _2}{2}\left| \sin \left( \frac{\theta _1-\theta _2}{2}\right) \right| \,d\theta _1d\theta _2 +O(\eta ^2). \end{aligned}$$

Because the time spent on a single ray is $\tau _k=d_k/v_0$, the expected search time in *N* steps is

46 $$\begin{aligned} \mathbb {E}[\tau \,|\,N]=\frac{1}{v_0}\mathbb {E}\left[ \sum _{k=1}^{N}d_k\right] =\frac{N}{v_0}\mathbb {E}\left[ d_{12}\right] . \end{aligned}$$

Finally, (38) gives the expected search time as

47 $$\begin{aligned} \mathbb {E}\left( \tau \,|\,Q_0\right)&=\sum \limits _{N=1}^\infty \mathbb {E}[\tau \,|\,N]P_n(Q_0)\\&=\frac{8S}{\pi ^2 v_0 \varepsilon }+\frac{\eta }{ v_0 \varepsilon } \left( \frac{1}{2\pi } \int \limits _{0}^{2\pi }\int \limits _{0}^{2\pi }\frac{\rho _1 + \rho _2}{2} \left| \sin \frac{\theta _1-\theta _2}{2}\right| \,d\theta _1d\theta _2 + \frac{32\pi }{\varepsilon } K_1(f_{\rho }) \right) \nonumber \\&\quad +O(\eta ^2). \nonumber \end{aligned}$$

This expansion is valid for $\eta \ll \varepsilon$. It shows that the first perturbation term depends on two integrals involving a generalized curvature function over the boundary. Although we shall not use directly these estimations in the numerical analysis below, these formulas reveal the geometrical features contributing to the search time. It would certainly be interesting to obtain a full expansion to order $\varepsilon ^n$.

## Search for a small target in a uterus-like geometry: a simulation approach

**Fig. 4:**
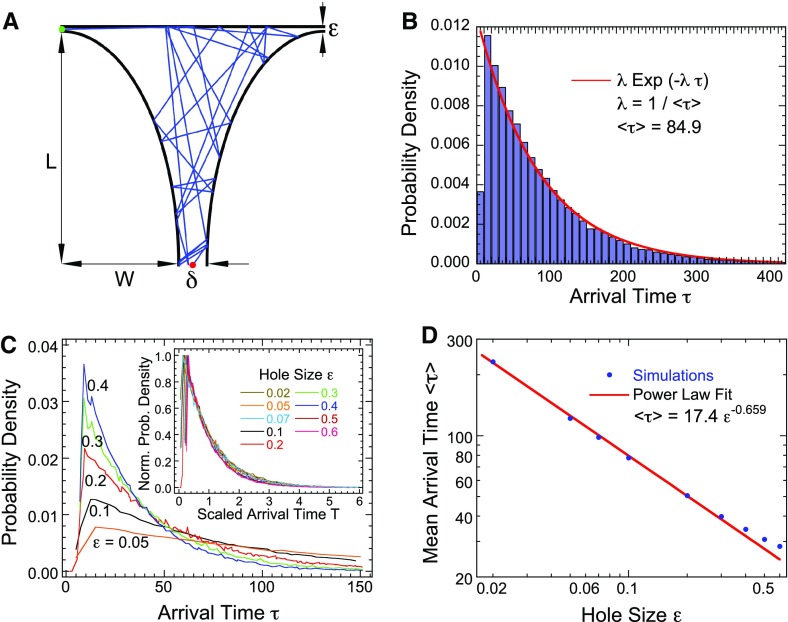
Search process in uterus-like geometry explored by simulations. **a** Schematic representation of a two-dimensional uterus-like domain containing a typical trajectory before reaching a small target. **b** Probability density distribution of the search time, when the target size $\varepsilon =1$ non dimensional unit ($L=5$, $W=2.5$). **c** Probability density distribution of arrival time for different target size $\varepsilon$ and the inset is the normalized pdf of arrival time normalized by the mean for various opening sizes $\delta$. **d** The mean arrival time in a uterus-like geometry decays approximately with $\mathbb {E}\tau ={C}{\varepsilon ^{-2/3}}$, where the target size is $\varepsilon$ and $C=17.4$

Consider the search problem for a small target in a two-dimensional non-convex domain with cusps (Fig. 4) and assume the dynamics (1)–(2). The two-dimensional domain in Fig. 4a captures some of the main features of the planar cross section of the uterine cavity (see discussion of the three-dimensional approximation below). The planar domain consists of two quarter-ellipses (black thick lines) underneath the upper part which is made of a straight line (black line on the top). The left and right sides are symmetric. The gaps between the straight line and the quarter-ellipses are the narrow openings.

### Estimate of the expected the search time from numerical simulations

The simulation of equations (1)–(2) with random reflections at the boundary, except when a trajectory hits one of the two ending cusps, where it is terminated, gives the results shown in Fig. 4b–d (see Appendix A.1 for the details of the simulations and Table 1: we use the following non-dimensional parameters $L= 5,W=2.5$, $\epsilon =0.1$, $\delta =0.3$ and velocity $v=1$). A typical trajectory before reaching a small target is shown in Fig. 4a. The probability density function (pdf) $\Pr \{\tau _{\varepsilon }=t\}$ of the arrival time to either one of the cusp opening (size $\varepsilon =0.1$) is shown in Fig. 4b. The tail of the distribution is well approximated by an exponential

48 $$\begin{aligned} \Pr \{\tau _{\varepsilon }=t\} \approx \exp (-\lambda t),\quad \hbox {for } t\gg 1, \end{aligned}$$

**Table 1:** Parameters for the arrival time simulations

Parameter	Description	Value
$\Omega$	Domain	$\Omega$
$\varepsilon$	Target size	
*W*	Width of the ellipse	
*L*	Height of the ellipse	
$v_0$	Measured in vivo velocity of spermatozoa	$75\, \mu \mathrm{ms}^{-1}$.

where the rate is $\lambda = \mathbb {E}[\tau _{\varepsilon }] ^{-1}$. The results of numerical simulations give the following value for the mean time $\mathbb {E}[\tau _{\varepsilon }] =84.9$. The exponential approximation for the tail seems valid for $t>100$, but deviates slightly when $20<t<100$. The short-time distribution is not studied here for $t<20$. We show the different pdfs as $\varepsilon$ varies in Fig. 4c. After rescaling the pdfs by their mean values, they all align into one universal curve (Fig. 4c, inset), suggesting that a possible power scaling law for the pdf. To explore this possibility, we estimated the search time for various values of $\varepsilon$ (Fig. 4d). Using a log-log plot, we compare the arrival time computed from stochastic simulations (blue dots) and the approximation by a power law (read line) $\mathbb {E}[\tau ] \sim C \varepsilon ^{-\alpha }$. An optimal fitting procedure shows that $\alpha = 0.658 \approx 2/3$. At this stage, we obtain the following approximation

49 $$\begin{aligned} \mathbb {E}[\tau ] \sim C \varepsilon ^{-2/3}. \end{aligned}$$

The power law is an approximation for small $\varepsilon$, but deviates as $\varepsilon$ becomes of order *O*(1) ($\varepsilon >0.2$ in Fig. 4e).

To convert in dimensional units, we use that a spermatozoa mean velocity is $v= 75\,\mu m s^{-1}$ (Reynaud K, private communication) and that for the length in Fig. 4 are $L= 7.5~\mathrm{cm}, W=2.5~\mathrm{cm}$, $\varepsilon =0.03~\mathrm{cm}, \delta =0.2~\mathrm{cm}$ (where the non-dimensional velocity was $v= 1$) and the non-dimensional arrival time was $\mathbb {E}[\tau ]\approx 238$ (Fig. 4). By scaling with the velocity, we obtain the dimensional search time $\mathbb {E}[ \tau _{DU}] =238\cdot 130= 30{,}940\,\mathrm{s}$, which is about 8.6 h. This two-dimensional estimate provides a time scale for the arrival to one of the two targets. Indeed, an egg is positioned at one of these places, from which we obtain the expected search time as $\mathbb {E}[ \tau _{target}] \approx 17$ h. This time estimation is compatible to previous experimental report (Chang 1951). Finally, the shortest time, which can be obtained by the concatenation the two straight lines (see discussion below) gives an estimate of 1333 s, which is about 22 min.

### Effect of changing some geometrical parameters on the search time

**Fig. 5:**
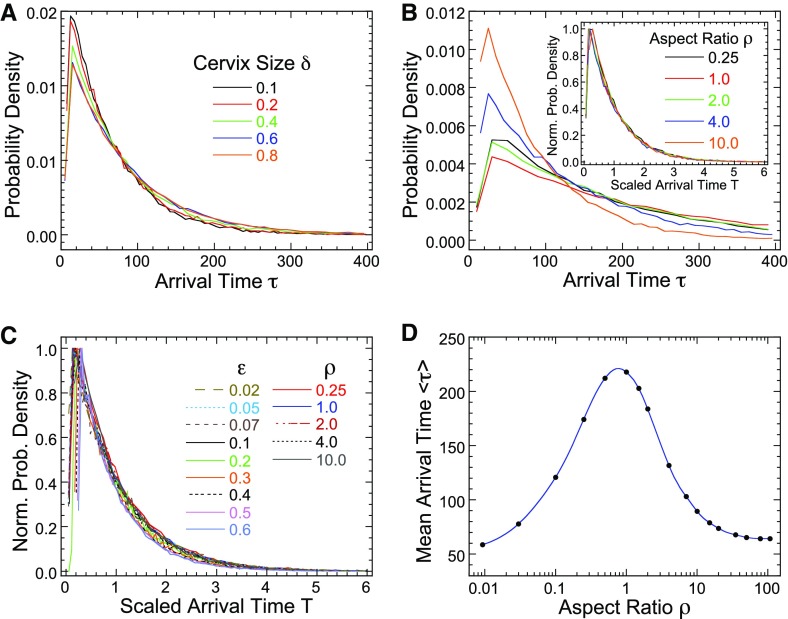
Exploring various parameters and the geometry of uterus-like domain. **a** Pdf of the search time for $\varepsilon =0.1$ for various values of the cervix size $\delta$. **b** Changing the aspect ratio $\rho =L/W$ affects the pdf of arrival time. The pdfs of the arrival time, normalized by the maximum are identical (*inset*). **c** Normalized pdfs for different parameters $\varepsilon$ and $\rho$. **d** Expected search time for various aspect ratios $\rho$. All fixed parameters are the same as in Fig. 4

We now explore the consequences of changing various parameters on the arrival time, this includes looking at the geometry of the uterus-like domain. We vary here several parameters such as the target size $\varepsilon$, the length *L* and the width *W* and the entrance size $\delta$, which represents the cervix outside radius. First, by varying the size $\delta$ (in range $[0.1-0.8]$), while keeping the other parameters constant (Fig. 5a), we obtain that the pdfs of arrival time show only a slight difference (less than 20 %). Thus, the numerical simulations show that the cervix size $\delta$ has little influence on the search time.

We next varied (Fig. 5b), the aspect ratio

50 $$\begin{aligned} \rho =\frac{L}{W}\in [0.25-10], \end{aligned}$$

as indicated in Fig. 5 (the length of the quarter of an ellipse *L* and *W* are defined in Fig. 4). For large aspect ratio $\rho$, the domain has a long narrow shape, while it is flat and wide for small aspect ratio. The pdf of the search time for different aspect ratios, while keeping the circumference of the quarter-ellipses fixed, is shown in Fig. 5b. Note that curves (normalized by the maximum) obtained for different parameters, align to a single curve (see inset of Fig. 5b), suggesting that the pdf has a scaling law structure. Actually, all normalized pdfs for the search time with different opening size $\delta$ and aspect ratios $\rho$ follow a universal curve (Fig. 5c), indicating that a general scaling law is expected as a function of the two parameters. Finally, we estimated numerically, the mean search time as a function of the aspect ratio (Fig. 5d): long narrow or flat geometries are associated with short mean search time, while a unit aspect ratio (a quarter-circle) has the maximum search time, four times larger. There is no theory today to explain this maximum.

## Discussion and conclusion

In this study, we presented the search process in a reduced domain presenting some aspects of uterus geometry, such as opening sizes of oviduct and cervix. We varied some parameters such as the aspect ratio $\rho =\frac{L}{W}$ defining the shape of the uterus and estimated the search time of a spermatozoon to the egg target site, in a cusp that represents the entrance of the oviduct.

In the absence of guide mechanisms, random reflection is the key element that determines the search time. We computed the expected search time in two- and three-dimensional balls and domains that resemble the uterus geometry. We found that the search time depends on the geometry of the domain. In two dimensions, we found for $\varepsilon \ll 1$ that

51 $$\begin{aligned} \mathbb {E}[ \tau _{\varepsilon }]=\frac{K S}{v_0\varepsilon }, \end{aligned}$$

where *K* is a constant, which we determined for a sphere and some convex domains. When the domain contains ends of cusp geometry,

52 $$\begin{aligned} \mathbb {E}[\tau _{\varepsilon }]=\frac{K_{C} S}{v_0\varepsilon ^{\alpha }}, \end{aligned}$$

where $K_{C}$ is now a dimensional constant. We could only obtain the exponent $\alpha =0.66$ numerically. The exact relation with the geometry remains an open question. The formula for a domain of arbitrary shape is unknown. Although the arrival time decreases with the oviduct opening size, the opening size of cervix (parameter $\delta$) had little effect. According to our numerical simulations, we found here that long narrow shape (high aspect ratio $\rho$) leads to arrival time much shorter than for a round uterus shape. These results should be considered as predictions because there are little available data on this subject.

The search time for the rectilinear motion studied here, describing spermatozoa motion, is much longer than for a diffusion particle, which is $O(\log \frac{1}{\varepsilon })$ in dimension 2 and $O(\frac{1}{\varepsilon })$ for a cusp (Holcman and Schuss 2013). We conclude that spermatozoa stay much longer in the domain before they can find the target, and when there are many swimmers, this long period can be seen as a selection process based on intrinsic spermatozoa properties, to select the fittest one. In three dimensions, we found that $\mathbb {E}[ \tau _{\varepsilon }] =\frac{K V}{v_0\varepsilon ^2}$ in a ball of volume *V*. We conjecture that this formula is valid for convex domains. However, for a domain with cusps, the scaling law may depend on the local cusp property. Another surprising result is that the power law for the search time in a cusp domain is smaller than 1, thus the search time is faster in a domain with a cusp compared to the disk. This situation is exactly the opposite for Brownian motion, where the search time becomes exponentially longer in a cusp (Schuss et al. 2007).

The present modeling approach allows for the first time to obtain laws for the expected search time. Most of the previously published numbers were obtained from empirical data, where spermatozoa were counted after opening the uterus at different stages (Chang 1951). Modeling is a different approach that accounts for the local motion and for the entire global geometrical structure of the uterus. Our approach should certainly be complemented by in vivo endoscopy studies. But there are still some difficulties in reconstructing the search process from the local data generated by the endoscopy probe that can only access local domains of few microns.

**Fig. 6:**
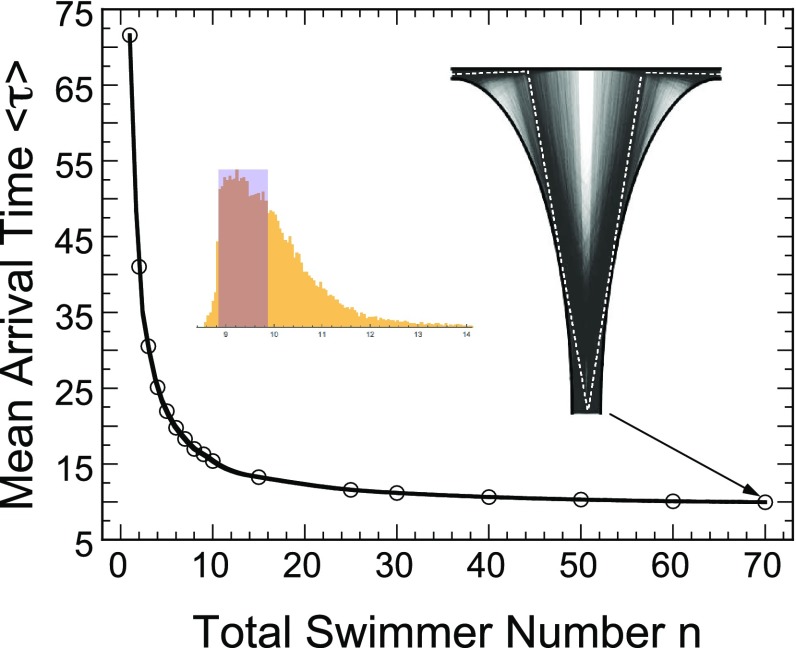
Optimal search paths. The optimal paths are approximated by the flow trajectories associated with short arrival time in the distribution. Trajectories associated with small arrival time are concentrated along two symmetric optimal paths (*white dashed line*)

Another consequence of the present analysis is that the number of swimmers matters when we consider the first one that finds a neighborhood of the egg. By increasing the number of trajectories (Fig. 6), when computing the arrival time of the first one, the trajectories that are almost optimizing the search time are located in a neighborhood around the optimal ones (white dashed lines in Fig. 6). The optimal trajectory is indeed the geodesic that joins the initial point to the final egg location. The shortest search time is achieved by the geodesic that minimizes the functional

53 $$\begin{aligned} \Lambda _{min} = \inf _{A_T} \int _0^T d(s)\,ds, \end{aligned}$$

where *d*(*s*) is the Euclidean distance at time *s* and

54 $$\begin{aligned} A_T =\{x: x(0)=x, x(T)=y, \quad \hbox {where } x \hbox { are piecewise constant trajectories} \},\qquad \end{aligned}$$

as indicated by the results of our simulations (Fig. 6). Possible future directions include the exploration of other parameters of the search process, such as the fitness of spermatozoa, the time window when they can sense the presence of the egg and chemotaxis processes, and so on. These elements should certainly be included in future models, as well as a possible killing field, which should account for sperm degradation during their sojourn time.

In summary, isometric shapes are not optimal for the search process and thus for fertilization. The uterus of most animals including human and rabbit, have long narrow shape, optimal for the search time and possibly fertilization, however, the optimization search process may also depend on other factors, such as body size, dynamics, surface of the uterus, uterus contraction and many more. The aspect ratio of the human uterus is around 2 to 3, which does not give the optimal ratio according to our simulations (Fig. 5) and we speculate that it might be associated with a lower fertility rate compared to other species.

## Figures and Tables

**Fig. 7:**
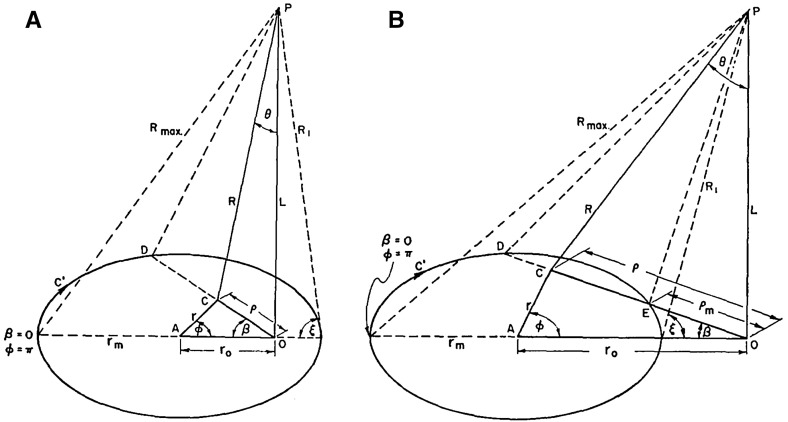
Solid angle subtended at points **a** over the interior or over the periphery of disk ($r_{0} \le r_{m}$); **b** outside disk boundary ($r_{0} > r_{m}$) (these figures are screenshots from Poxtan’s paper; Paxton 1959)

**Fig. 8:**
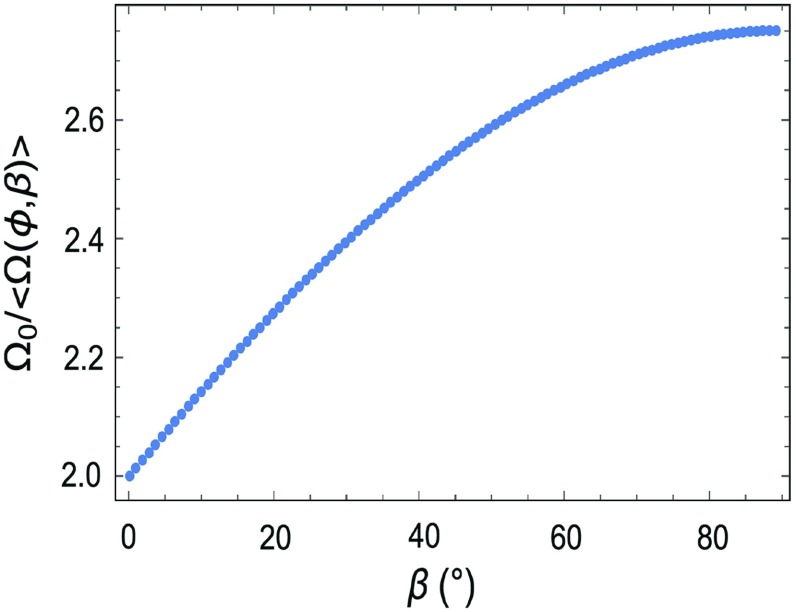
Ratio $\Omega _{0} / \langle \Omega (\phi , \varepsilon )$ computed numerically by using Eqs. 62–63

## Appendix

### A.1 Simulation procedure

In our numerical simulations on a uterus-like domain, trajectories always start at the bottom and moves according to Eqs. (1)–(2) inside the domain until the boundary, where trajectories are reflected with a random angle with respect to the tangent. In case of the uterus-like domain, there are two small targets located at the end of the two symmetrical cusps. The target can only be found by trajectories moving inside de the domain.

The escape time of a trajectory from the domain is computed from the total length before exiting, as the velocity is constant. For each case, the statistic is obtained for 30,000 runs. We did not see any differences by using additional simulations such as 50,000.

### A.2 Probability that a trajectory enter the solid angle of the target in the three dimensional ball

In this section, we compute the solid angle $\Omega (\phi ,\varepsilon )$ starting from any point $(\phi , \theta )$ located on a sphere. The solid angle subtended by a circular disk (Paxton 1959) is summarized here.

By definition, the solid angle is given by the integral

55 $$\begin{aligned} \Omega = \int \frac{\mathbf {n}_{P} \cdot \mathbf {ds}_P}{R^{2}}, \end{aligned}$$

where $\mathbf {n}_P\cdot \mathbf {ds}_P$ is the area of the projection of the surface element $ds_P$ onto the plane perpendicular to the radius $R=|NP|$, from the north pole to point P (see Fig. 7). Specifically, Integral 55 can be written as:

56 $$\begin{aligned} \Omega =\int \frac{ds \cos \theta }{R^{2}}=\int \int \frac{\rho d\beta d\rho \cos \theta }{R^{2}}=\int \int \sin \theta d\theta d\beta \end{aligned}$$

in which $\rho =L \tan \theta$ and $L/R=\cos \theta$. When $r_{0} \le r_{m}$ (see Fig. 7a), Eq. 56 can be written as

57 $$\begin{aligned} \frac{\Omega }{2}=\int _{0}^{\pi }\int _{0}^{\theta _{s}}\sin \theta d\theta d\beta =\pi -\int _{0}^{\pi }\cos \theta _{s}d\beta \end{aligned}$$

After a few more steps and re-arrangements, Eq. 57 turns into

58 $$\begin{aligned} \Omega = 2 \pi - \frac{2L}{R_{max}} K\left( k \right) - \pi \Lambda _{0} \left( \xi , k \right) \end{aligned}$$

where *K*(*k*) and $\Lambda _{0}(\xi , k)$ are the complete elliptic integrals of the first and third kind, respectively with

59 $$\begin{aligned} k^{2}= & {} 1-(R_{1}/R_{max})^{2} \nonumber \\ R_{1}^{2}= & {} L^{2}+(r_{0}-r_{m})^{2}\\ \xi= & {} \sin ^{-1}(L/R_{1}).\nonumber \end{aligned}$$

When $r_{0}>r_{m}$ (see Fig. 7b), Eq. 56 can be written as

60 $$\begin{aligned} \frac{\Omega }{2}=\int _{0}^{\beta _{max}}\int _{\theta _{m}}^{\theta _{s}}\sin \theta d\theta d\beta =\int _{0}^{\beta _{max}}\cos \theta _{m} d\beta - \int _{0}^{\beta _{max}}\cos \theta _{s} d\beta \end{aligned}$$

Similarly, after certain steps and re-arrangements, Eq. 60 can be written as follows:

61 $$\begin{aligned} \Omega (\xi , k) = -\frac{2L}{R_{max}}K\left( k \right) + \pi \Lambda _{0}\left( \xi , k \right) \end{aligned}$$

In summary, the expression for the solid angle is

62 $$\begin{aligned} \Omega (\xi , k) = \left\{ \begin{array}{ll} 2 \pi - \dfrac{2L}{R_{max}} K\left( k \right) - \pi \Lambda _{0} \left( \xi , k \right) , &{} r_{0}<r_{m} \\ \\ \pi -\dfrac{2L}{R_{max}}K\left( k \right) , &{} r_{0}=r_{m} \\ -\dfrac{2L}{R_{max}}K\left( k \right) + \pi \Lambda _{0}\left( \xi , k \right) , &{} r_{0}>r_{m}. \end{array}\right. \end{aligned}$$

$\Omega (\xi , k)$ is a function of $\varepsilon$ and $\phi$ as we shall see now. We apply formula 62 to the case of a small hole of radius $\varepsilon$ located at the north pole of the three-dimensional sphere. We obtain for a point of coordinates $(\theta ,\phi )$

63 $$\begin{aligned} R^2_1= & {} R^2(1-\cos \phi )^2+(R \sin (\phi )-\varepsilon )^2 \end{aligned}$$

64 $$\begin{aligned} R^2_{max}= & {} R^2(1-\cos \phi )^2+(R \sin (\phi )+\varepsilon )^2\end{aligned}$$

65 $$\begin{aligned} k^2(\phi )= & {} \frac{4\varepsilon R \sin (\phi )}{2R^2(1-\cos \phi )+2 \varepsilon R \sin (\phi ) +\varepsilon ^2}\end{aligned}$$

66 $$\begin{aligned} \sin (\xi )= & {} \frac{R(1-\cos \phi )}{R^2(1-\cos \phi )^2+(R \sin (\phi )-\varepsilon )^2}. \end{aligned}$$

We then used Mathematica to compute the $\int _{\phi >\varepsilon /R}\Omega (\phi )\sin (\phi )d\phi$. We obtain for small $\varepsilon$ that

67 $$\begin{aligned} \int _{\phi >\varepsilon /R}\Omega (\phi )\sin (\phi )d\phi =\pi \left( 1-\cos \varepsilon \right) . \end{aligned}$$

Finally, we show in Fig. 8 that $\beta \ll 1$, the ratio $\Omega _{0} / \langle \Omega (\phi , \varepsilon ) \rangle \approx 2$ for small $\beta$,
